# Cooperation Improvement in an Integrated Healthcare Network: A Social Network Analysis

**DOI:** 10.5334/ijic.6519

**Published:** 2023-06-26

**Authors:** Nicolás Larrain, Sophie Wang, Tom Stargardt, Oliver Groene

**Affiliations:** 1 OptiMedis AG, Hamburg, DE; 2 Hamburg Centre for Health Economics (HCHE), University of Hamburg, Hamburg, DE; 3 University of Witten/Herdecke, Witten, DE

**Keywords:** Social Network Analysis, integrated healthcare systems, physician cooperation, dynamic panel analysis

## Abstract

**Background:**

Cooperation is a core feature of integrated healthcare systems and an important link in their value-creating mechanism. The premise is that providers who cooperate can promote more efficient use of health services while improving health outcomes. We studied the performance of an integrated healthcare system in improving regional cooperation.

**Methods:**

Using claims data and social network analysis, we constructed the professional network from 2004 to 2017. Cooperation was studied by analyzing the evolution of network properties at network and physician practice (node) level. The impact of the integrated system was studied with a dynamic panel model that compared practices that participated in the integrated system versus nonparticipants.

**Results:**

The regional network evolved favourably towards cooperation. Network density increased 1.4% on average per year, while mean distance decreased 0.78%. At the same time, practices participating in the integrated system became more cooperative compared to other practices in the region: Degree (1.64e-03, p = 0.07), eigenvector (3.27e-03, p = 0.06) and betweenness (4.56e-03, p < 0.001) centrality increased more for participating practices.

**Discussion:**

Findings can be explained by the holistic approach to patients’ care needs and coordination efforts of integrated healthcare. The paper provides a valuable design for performance assessment of professional cooperation.

**Highlights:**

- Using claims data and social network analysis, we identify a regional cooperation network and conduct a panel analysis to measure the impact of an integrated care initiative on enhancing professional cooperation.
- Physician practices participating in the integrated system became more cooperative and improved their influence in the regional network more than non-participating practices.
- Integrated healthcare systems effectively incentivize cooperation through a holistic approach to patient care needs and coordination efforts.

## Introduction

Integrated healthcare systems (IHSs) are defined by value-based principles and seek to shift the focus of healthcare from a supply-led to an outcomes-led approach [1]. In practice, IHSs address care fragmentation by organizing provider networks, bolstering investment in prevention and health intelligence while improving quality of life and avoiding unnecessary costs. IHSs have had documented success in achieving positive economic, care experience and population health outcomes [2,3,4].

Given patients’ multiple and ever-growing needs, the search for improving system efficiency puts pressure on multi-professional teamwork between healthcare providers [5,6]. Consequently, good professional teamwork, in the form of collaboration, coordination and cooperation between care providers is constantly fostered in IHSs. These concepts differ from each other. Collaboration and coordination imply structure, such as in implementing a care pathway or adopting protocols in a hospital unit [7]. Conversely, cooperation happens by actively sharing clinical information through informal discussions and observations [8]. IHSs understand that providers who excel at these concepts can promote more efficient care services while improving health outcomes [9]. In line with the value-based principles of IHSs, this premise is supported by extensive literature linking professional relationships with health system performance.

In 1986, Knaus [10] supported the idea that the level of coordination of intensive care units significantly influences their effectiveness. Several authors more recently highlighted the positive association between professional relationships and patient outcomes. Netting and Williams [11] support attending care providers’ professional relationships to improve patient outcomes and provider satisfaction. Cunningham et al. [12] studied the effects of relationship network structure and concluded that cohesive and collaborative health professional networks contribute to improving care quality and safety.

Fattore et al. [13] described two lines of thought to explain why primary physicians working in collaborative arrangements have similar professional behaviours. First, the “social capital framework” states that individuals acquire information and other resources through professional relationships. Second, the “social influence framework” says that individuals influence one another through professional relationships and that these connections act as avenues of information transfers for developing and enforcing social norms. Fattore’s work is supported by the findings of Nair et al. [14] that prescribing behaviours are influenced by key opinion leaders, defined by having an advantageous position in the professional network.

The social influence and social capital frameworks provide the basis for using Social Network Analysis (SNA) to represent the value of professional network positioning and to study its association with performance. SNA studies social structures using networks and graph theory [15]. By characterizing the network structure in nodes (actors of the system) and edges (relationships between actors), SNA emphasizes both the importance of the individual and the channels of information exchange, social influence and other resources [16]. Network parameters derived from SNA have been used as predictors of performance indicators in a wide array of sectors. For example, SNA-derived parameters are influential in the performance of information technologies [17], knowledge sharing [18], job performance in 5 different production sectors [19], the performance of scholars [20] and innovation and performance of organizations in various industries [21]. In the healthcare sector, SNA-derived parameters of professional networks have shown significant influence on hospitalizations and readmission rates [22], hospitalization costs [22], patient satisfaction [23], length of stay [24] and mortality [10], among others [25].

Considering the extensive literature supporting the positive effect of professional cooperation to improve health system performance, IHSs understand cooperation relationships as an essential step in their value-creating mechanism [6,26]. For this reason, and in line with the Donabedian approach for performance assessment (structure-process-outcome) [27], monitoring cooperation is a crucial process-level assessment in need of a reliable assessment method.

However, the lack of specific tools to measure the core functions and value-creating mechanisms of IHSs is well documented [2,28,29]. Physician cooperation is notably overlooked, as no indicators assessing physician cooperation are presented by countries or experts evaluating IHSs in the EU [2,28,29]. Previous assessment methods that address this gap used surveys of patients and providers to study cooperation [30]. However, surveys are expensive to conduct periodically and lose accuracy quickly when trying to signal past events. Hence, they are not adequate for monitoring cooperation systematically.

An alternative method is described by Barnett and colleagues [8,31]. As described by the authors, because of the connection between doctors they represent, shared patients “arising because of referral, patient self-selection, administrative rules, or even chance” [31], present an opportunity to be a proxy for meaningful information-sharing physician relationships. Given that shared patients can be used to discern cooperation relevant to patient care among pairs of physicians, they can be used to define organic physician networks [32,33]. DuGoff et al. [25] reviewed 49 papers where authors construct networks based on shared patients. These networks reflected aspects of professional teamwork and were studied in various settings, including countries, states, hospital referral regions, or within a hospital unit. In these networks, the strength of each relationship is determined by the number of shared patients [8]. The authors highlight that identifying a meaningful information-sharing relationship among the patient sharing relationships is a key consideration for recreating a professional network correctly. Authors use mainly two methods for this purpose. One is having a strict threshold of shared patients, after which one can assume a meaningful information-sharing relationship. The alternative consists in classifying as meaningful the relatively strongest patient-sharing relationships. The relative method will necessarily reflect exploratory analysis, while the absolute method can be determined a priori and used in causal analysis [25].

Our article studies the professional network of the Kinzig river valley region in Germany and uses the case of “Gesundes Kinzigtal” (GK) to create a framework for systematically evaluating the evolution of regional cooperation and to study the extent to which the effect on cooperation is attributable to integrated healthcare. The network is constructed using SNA, based on yearly shared patients for 14 years. The evolution of cooperation is measured at two levels of analysis – at the network level, with descriptive statistics and at node level, in a dynamic panel analysis. Indicators for each level are drawn from the network’s structural parameters.

## Background

Gesundes Kinzigtal is an IHS operating in the Kinzig river valley, a semi-isolated region in the south of Germany. The system brings together a local network of physicians, a professional health management company called OptiMedis AG, and various other health-related organizations such as social health insurance, patients’ associations, gyms and pharmacies. The system is supported by a long-term shared savings contract and is accountable for the care of around 35 000 people, more than half of all the people living in the region. The network is managed by a regional integrator (Gesundes Kinzigtal ltd), an entity created for this purpose and to implement system interventions [3]. The generated savings are shared between the health insurance and the integrated system, which in time finances the regional integrator and reinvests in health intelligence, performance bonuses and other value-based interventions. Physicians in the region are free to join the integrated system. Likewise, patients in the region can enrol directly with the regional integrator. This way, GK has two work streams to achieve its objectives. Enrolled patients have access to special care programs, case management and other interventions to improve care efficiency and promote healthy lifestyles. Partner physicians receive health intelligence support and participate in interventions to improve care integration, such as clinical practice alignment and performance evaluation. In line with the core values of integrated care, cooperation between providers is constantly fostered in GK. The integrated system has had documented success in achieving cost savings and increasing population health [3,4] and is seen as a best practice in integrated healthcare implementation [34,35].

GK started organizing the provider network in 2004. The free participation of outpatient physician practices in the region allows us to study the effects of integrated healthcare by comparing the evolution in cooperation of physician practices that participate (referred to as “integrated practices” or “IPs” for short) to other physician practices in the region (“Non-IPs”).

Being an IP shows that the practice values the integrated approach and understands its benefits, including those derived from improving professional cooperation. Second, while being part of the integrated system, IPs are exposed to several value-based interventions intended to obtain and potentiate the effects of the integrated approach, including monetary incentives such as performance bonuses derived from the shared savings contract. We assume that the effect of IPs’ intrinsic motivation to cooperate affects the evolution of their cooperation through, and only through, the integrated system. Under this assumption, the self-selection of IPs to join the integrated system doesn’t affect the effect estimation of being an IP. The assumption is plausible because GK was born partly from the intrinsic motivation to cooperate of a group of physicians in the region. Consequently, the operations of the integrated system were built (among other things) around exploiting, incentivizing and enhancing the benefits of said motivation to cooperate.

## Methods

### Data

Data for the analysis included all the claims of (pseudonymized) outpatient physicians in the Kinzigtal region; from 2004 to 2017 for patients from one statutory health insurance. This corresponds to around 50% to 55% of all the patients in the region. Outpatient physicians include both general practitioners and specialists. The unit making the claims corresponds to physician practices, where in some cases, one claiming unit includes more than one physician. This being said, it is common for outpatient physicians to have a personal practice, especially in the rural context [36].

Complementing physician practice claims with data from hospitals, prescriptions, and other claims, we were able to construct a medical history of each pseudonymized patient. Furthermore, data contains the identification of the claim as an emergency or routine patient visit, and it is possible to identify if physician practices correspond to general practitioners or another speciality.

### Network creation

Following the method described by Barnett and colleagues [8,31], our network positions physician practices as nodes and meaningful cooperation relationships as edges connecting the nodes. Barnett et al. asked physicians about their cooperation relationships in a survey and compared the resulting cooperation network to the one defined by shared patients [31]. They found that pairs of physicians with more shared patients were more likely to report having a meaningful professional relationship. By comparing the survey-based cooperation network to networks created based on different thresholds of shared patients, the authors defined the sensitivity and specificity of the shared patients indicator for identifying a meaningful information-sharing relationship. The analysis showed that the probability of finding a real information-sharing relationship plateaus at 9 or more shared patients. Studies using this method within a hospital setting use lower thresholds of shared patients to indicate a meaningful relationship (2 or more in Pollack et al [9]; 3 or more in Barnett et al. [31]). On the other hand, in a setting more closely related to the one of GK, Landon et al. [8] use 8 shared patients or more to identify a meaningful information-sharing relationship between physician practices in a hospital referral area in the US. We took a conservative approach and used 9 shared patients or more to identify a meaningful cooperation relationship. This threshold identifies meaningful information-sharing relationships 82% of the time [31]. In other words, only 18% of physician pairs that share 9 or more patients don’t have an actual information-sharing relationship. The sensitivity of our results to the threshold of 9 or more patients was tested by replicating the analysis using 15, 12, 6, and 3 or more shared patients as thresholds, in line with previous literature [8,9,25,31].

Emergency visits and visits to Anaesthesiology, Radiology and Neuropathology were excluded. The exclusion follows the methods of previous literature [25,31] and responds to the fact that these visits have little physician-patient contact or do not participate in the patient’s care coordination. Finally, practices that provided care to less than 30 patients a year are also excluded, as it is assumed that they do not actively participate in the regional network [33].

We converted the database into an adjacency matrix for network analysis [37]. The adjacency matrix is unipartite and represents the number of patients shared by pairs of practices. The process is further explained in appendix 1. The network was created for each year using the “network” package in R.

### Outcomes

We studied 5 outcomes, two at network level and three at node level. At network level, we first calculated network density as the total number of edges divided by the total number of possible edges if all the nodes were connected to each other. In essence, it is a measure of cohesiveness, indicating the level of connectivity in the network [25]. Network density tells us the number of existing cooperation relationships relative to the number of nodes, with a denser network indicating a more cooperative network. Network mean distance is the average number of edges in the shortest path between any two nodes. Mean distance tells us the average distance of any node to another node. A smaller mean distance shows that the paths of information flow are faster, indicating more efficient information transfers in the professional network.

A higher density implies a lower mean distance by construction. To study the evolution of mean distance independently of network density, we used random graph models [38] to create an independent indicator of mean distance. The method generates random graphs with the same density and number of nodes as the observed network and recreates the distribution of potential mean distances with these characteristics. Then, it measures the distance in standard deviations between the observed mean distance and the average of the potential mean distances generated with the random graphs to understand how extreme the observed value is. By comparing how extreme the observed mean distance is in the different years, we can evaluate if the network became more efficient in time, independent of the changes in density. We called this new indicator “Transformed mean distance”.

At node level, we studied three indicators of centrality. Centrality measures a node’s ability to send, receive or interrupt information flow [25] and determines the importance of a node in the network by focusing on the immediate subnetwork formed by the nodes connected to itself. **Degree centrality** (Degree) is calculated as the number of edges of each physician practice to its peers. Intuitively, more meaningful cooperation relationships are indicative of a more cooperative practice. **Eigenvector centrality** (EV-centrality) extends the measure of node importance by accounting not only for the number of edges, but also the centrality of the nodes the node in question is connected with. The score is based on the concept that connections to highly connected nodes are worth more than connections to less connected nodes [39]. EV-centrality indicates the node’s influence in the professional network, whereas higher EV-centrality indicates more influence. However, the measure is arbitrary and only comparable within the same network. This makes it not comparable in time for changing networks. To address this shortcoming, we created an EV-centrality percentage ranking per year and evaluated the evolution of the node’s position in the ranking in the years of assessment. **Betweenness centrality** (Be-centrality) is measured as the proportion of pathways in the network where the node of interest is on the shortest path between any two nodes. This measure indicates the node’s importance as a catalyst of information transfers. As with EV-centrality, we created a percentage ranking to compare the evolution of this centrality over time. A higher position in the ranking will indicate that the node contributes more to the network’s efficiency in information transfers.

### Analysis

#### Network level

At network level, we analyzed the evolution of the cooperation indicators with descriptive statistics and obtained the average percentage changes in network density, mean distance, transformed mean distance, number of physician practices, number of IPs, and total number of patients in the network. A time series analysis was considered but discarded because of the small number of observations (fourteen) and the impossibility of including relevant covariables at network level.

#### Node level

At node level we considered the stability of cooperation relationships over time, hence we analyzed the evolution of the cooperation indicators with a (unbalanced) dynamic panel analysis with fixed individual effects. Equation 1 represents the dynamic panel data model with time *t* and individuals *i*. Where *y* represents any of the cooperation indicators at node level, and *y_i,t–z_* is the z^th^ lag of *y*. Predictors of interest correspond to the year (*year*) as a numeric term and a combination of a dummy variable indicating if the practice was part of the integrated system (*IP*) and the year variable. To control for the size of the practice and, indirectly, the number of physicians per practice, we used the total number of patients seen per year (*Nº patients*). Likewise, to control for the level of sickness of a physician’s patients, we included the mean Charlson comorbidity score (*Charlson*) of all the patients seen in each practice that year. Finally, we added individual fixed effects to control for constant variables (observables and unobservable) related to each practice (*FE*) that could influence the prediction of the network indicators, like physician speciality or geographical location. The fixed effects absorb all the node characteristics that remain unchanged in the time frame for analysis, including the participation in the integrated system (*IP*) and the intrinsic motivation that might influence cooperation. Random effects were discarded, as it’s plausible to think that individual-specific effects are correlated with the independent variables [40]. This was confirmed with the Durbin–Wu–Hausman test. The estimator of the combination term (*θ*_3_) allows us to test if the evolution of the indicators among practices participating in the integrated system was different than for the rest of the network.

Equation 1.

\begin{array}{l}
{y_{it}} = {\theta _1}{y_{i,t - 1}} + \,{\theta _2}yea{r_{it}} + {\theta _3}I{P_i}yea{r_{it}} + {\theta _4}N^\circ patient{s_{it}}\\
\,\,\,\,\,\,\,\,\,\,\,\,\,\, + {\theta _5}Charlso{n_{it}} + F{E_i} + {\varepsilon _{it}}
\end{array}

We analyzed the dynamic approach validity by testing the first and second order autocorrelation. After confirmation, we used a method that consistently estimated the estimators *θ* considering the endogeneity generated by the correlation between *y_i,t–_*_1_ and the error term(*ε_it_*) during the demeaning^1^ process for calculating fixed effects [41]. We used a two-step system Generalized Method of Moments (GMM) estimation as defined by Blundell and Bond [42] to estimate equation (1.) for our cooperation indicators. The method uses further lags as instruments of the predictor lagged variable(s) in equation 1. The appropriateness of the instruments was validated with a Hansen-Sargan test [43]. Goodness of fit was measured calculating the square correlation between the predicted cooperation indicators and the observed indicators. This measure is equivalent to R-squared for OLS regression [44]. The Dickey-Fuller test was used to check for stationarity. Multicollinearity, normality in error distributions, heteroskedasticity and influential observations were also assessed. Calculations used R package pgmm. Further specification of the estimation method can be found in appendix 2.

## Results

### Network level

The professional network in the region experienced substantial changes during the years of analysis. Over the 14 years, 602 different physician practices participated in the regional network, on average having 8.76 (standard deviation 4.1) years active in the region. Table 1 summarises the results of the network level analysis. The network level indicators presented roughly constant changes in time. The total number of patients increased from 25 979 in 2004 to 29 421 in 2017 (average yearly increase of 0.97%). The number of active physician practices per year also increased, from 197 in 2004, to 225 at the end of the assessment, with an average yearly increase larger than that for the patients (1.15%). Meanwhile, IPs increased from 28 in 2004 to 36 in 2017.

**Table 1:** Measures for network-level indicators of cooperation.


YEAR	DENSITY	MEAN DISTANCE	TRANSFORMED MEAN DISTANCE INDICATOR	Nº OF PRACTICES	Nº OF INTEGRATED PRACTICES*	TOTAL NUMBER OF PATIENTS

2004	0.136	2.089	35.03	197	28	25,979

2005	0.151	2.020	36.60	192	29	26,491

2006	0.137	2.007	29.00	217	37	27,360

2007	0.139	1.995	25.73	209	33	27,621

2008	0.144	1.922	16.81	225	37	27,209

2009	0.151	1.933	24.77	221	35	27,408

2010	0.161	1.893	17.30	218	35	27,306

2011	0.157	1.879	10.61	222	35	27,804

2012	0.144	1.923	18.19	229	34	28,032

2013	0.150	1.888	10.97	228	34	28,612

2014	0.153	1.877	8.82	230	36	28,151

2015	0.162	1.881	14.25	220	35	28,487

2016	0.152	1.890	13.86	234	36	28,999

2017	0.160	1.884	14.73	225	36	29,421

Average percentage change	1.40%	–0.78%	–0.50%	1.15%	2.32%	0.97%


* Integrated practices correspond to physician practices participating in GK. (IPs).

**Figure 1:**
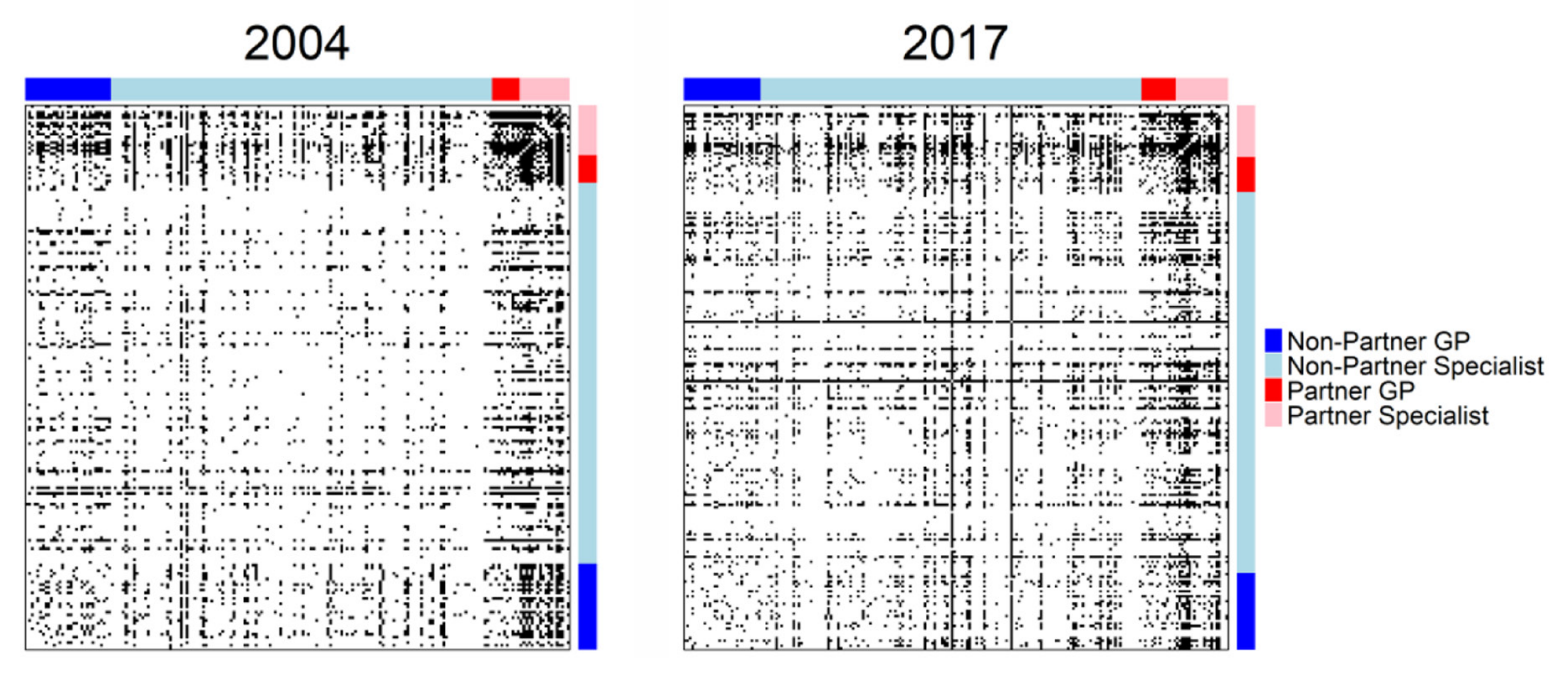
Regional professional network. IPs vs Non-IPs differentiated by GPs and other specialties.

Figure 1 presents two heat maps representing the cooperation network. All physician practices are displayed horizontally and vertically, and each black pixel represents a meaningful cooperation relationship between each pair of practices. The type of physician in each axis is represented by colour. Red represents participating general practitioners, pink represents participating specialists, blue represents non-participating general practitioners, and light blue represents non-participating specialists. The darkening observed between 2004 and 2017 shows cooperation increases in the regional network. The enhanced opacity of the areas associated with practices participating in the integrated system shows their leading role in improving cooperation.

Edge density rose from 13.6% in 2004 to 16% in 2017, with an average yearly increase of 1.4%. This increase in density shows a more cohesive and cooperative network. The mean distance between any two nodes decreased from 2.09 in 2004 to 1.88 in 2017. This means information in the network flowed faster than when integrated healthcare started. To create the transformed mean distance indicator, we generated 5 000 random graphs with the observed density and number of nodes per year. The distance (in standard deviations) of the observed mean distance from the randomly generated graphs’ average mean distance diminished from 35.03 to 14.03 (average yearly decrease of 0.5%). This indicates a more efficient network independent of the changes in density.

### Node level

At the start of the assessment, the average degree for IPs was 60.41 cooperation relationships. For their Non-IP peers, the mean degree was 25.34. Similarly, the EV-centrality ranking of IPs was on average in the 77.8 percentile, while Non-IPs were on average at the 45.4 percentile. This tendency continued to the BE-centrality ranking (73.3 vs 46.21).

Our dynamic approach proved adequate as all cooperation indicators’ models presented significant serial correlation. This is not surprising, as cooperation relationships are stable bonds meant to last for several years. Table 2 shows the results for indicators estimated with system GMM. The Sargan tests (null hypothesis: The instruments as a group are exogenous) confirm the validity of the instruments in all models (p values > 0.05) [45]. At the same time, the test for autocorrelation of second order fails to reject the null hypothesis of no second-order serial correlation (p values > 0.05). This implies that the original error term is serially uncorrelated, and the moment conditions are correctly specified.

**Table 2:** System GMM models.


	MODEL 1	MODEL 2	MODEL 3
		
	DEGREE	EV-CENTRALITY RANKING	BE-CENTRALITY RANKING
		
	COEFFICIENTS (SE)	COEFFICIENTS (SE)	COEFFICIENTS (SE)

lag(y, 1)	0.52***	0.59***	0.32***
	(0.12)	(–0.12)	(0.05)

lag(y, 2)	0.04		
	(0.14)		

year	1.70e-04	3.96e-03*	1.05e-02***
	(5.39e-04)	(1.65e-03)	(1.21e-03)

year*IP	1.64e-03·	3.27e-03·	4.56e-03***
	(9.08e-04)	1.82e-03	(7.94e-04)

Charlson Score	1.73**	2.89***	2.31***
	(0.61)	(0.78)	(0.61)

Total Nº patients	1.93e-02*	0.01	1.48e-02***
	(8.83e-03)	(5.38e-03)	(2.01e-03)

#Obs	3067	3067	3067

nodes	602	602	602

Length of res. vector	4493	3675	4894

Sargan test:	chisq(68) 58.95 (p-value: 0.78)	chisq(52) 67.45 (p-value: 0.08)	chisq(81) 74.91 (p-value: 0.67)

Autocorrelation test (1)	normal –1.98 (p-value: 0.04)	normal –5.28 (p-value: 1.32e-07)	normal –6.15 (p-value: 7.89e-10)

Autocorrelation test (2)	normal 1.04 (p-value: 0.30)	normal 1.89 (p-value: 0.06)	normal 0.91 (p-value: 0.37)

Wald test for coefficients	chisq(6) 7946.341 (p-value: < 2.22e-16)	chisq(5) 16151.79 (p-value: < 2.22e-16)	chisq(5) 10526.45 (p-value: < 2.22e-16)

R2 *corr*(*y, ŷ*)^2^	0.89	0.92	0.83

Instruments	Lags 4:11	Lags 6:11	Lags 3:14


Significance: *** = p < 0.001; ** = p < 0.01; * = p < 0.05; · = p < 0.1; SE = Standard Errors.

In the estimation of degree in model 1 of Table 2, the combination variable of the IP participation and the time variable (1.64e-03, p = 0.07) tell us that practices that took part in the integrated system increased their cooperation relationships more than physician practices who did not participate (at 10% error). The average Charlson score (1.73, p < 0.01) and total number of patients (1.93e-02, p < 0.05) present plausible significant coefficients as sicker patients will need more doctors and it is more likely that larger practices will share more patients.

The positions in the EV-centrality percentage ranking changed considerably over the years. The mean standard deviation of position changes per practice was 13.4 positions, while the mean difference from first to last position was +1.8. The positive significant coefficients of the “*year*” predictor in model 2 of Table 2 (3.96e-03, p < 0.05) point towards a positive evolution of practices’ influence in the network. The positive and significant (at 10% error) coefficient of the combination variable (3.27e-03, p = 0.06) tells us that practices that are part of the integrated system increased their influence position more than their non-integrated peers. Similarly, the “*year*” variable (1.05e-02 p < 0.001) in model 3 of Table 2 tells us that with time all practices become more important information transfer catalysts. At the same time, the combination variable (4.56e-03, p < 0.001) tells us that IPs became more important in accelerating information flows in the network. The control variables behaved similarly in all models of Table 2. Assumption testing is provided in appendix 3. The inclusion or exclusion of the nodes with the most leverage in the different models did not change the conclusions of the analysis.

### Sensitivity analysis

We tested our models with a threshold of 15, 12, 6 and 3 or more patients to identify a meaningful cooperation relationship. Using 6, 12, and 15 patients, our assessment of network density and network mean distance showed similar results. Using 3 patients, the assessment for network density shows similar results, but the mean distance showed nonconstant changes.

The sensitivity analysis results for the node-level model can be found in appendix 4. The direction of the effects was not affected by choice of the threshold. However, the significance of the coefficient *θ*_3_ of the variable of interest *IP_i_year_it_* was affected when using the most extreme values (3 and 15) in the model for degree centrality. A similar phenomenon occurred in the model for BE-centrality (although the effects remained significant at 5% error). This is a plausible result given the plateau evidenced by Barnett and colleagues [8,31] at 9 shared patients and the low specificity of lower thresholds. The interest variable in the EV-centrality model remains significant for 9, 12(p-value 0.10) and 15 but loses significance for lower thresholds.

## Discussion

The evolution of regional cooperation was favourable in the years of analysis. Further, the positive evolution was led by physician practices participating in the integrated system. Density increased on average every year. Similarly, independently of the changes in density, the mean minimum distance between any two nodes decreased. The potential influence of the increasing number of patients over network density and mean distance is controlled by the increase in the number of doctors and doctor-to-patient ratio. This is a trend in line with national reality [46].

At node level, even though IPs had more cooperation relationships at baseline, their increase in cooperation relationships was significantly larger (at 10% error). Similarly, even though IPs were in the top 25% of both centrality rankings at the beginning of the assessment, they consistently moved to a more cooperative position. Combined, these effects suggest extra efforts from IPs to improve regional cooperation.

Control variables behaved in line with expectations, highlighting the positive and significant effect of the *“year”* variable in both models using centrality rankings, but unsignificant effect in the model for degree centrality. Two effects can explain this phenomenon. One is the higher influence and importance achieved over time for any practice in the network because of experience. The other is the low EV and BE centrality of new practices that are added to the network, pushing old practices upwards in the ranking.

Even though significant, effects sizes for the variable *IP_i_year_it_* are small. Considering the advantageous position of practices participating in the integrated system at baseline, this finding can be explained by plausible diminishing marginal returns of creating cooperation relationships and maintaining important and influential positions. In other words, we think it is plausible that more cooperative practices benefit less from expanding their cooperation relationships further. We can expand this idea and hypothesize that a physician can benefit from a limited number of cooperation relationships, after which the benefit of the next cooperation relationship will be less than its cost. This limit could be related to the spectrum of medical specialities, for example. Further qualitative research is needed to enlighten this topic.

Considering initial conditions, we can be certain that there is a self-selection of more cooperative practices to participate in integrated healthcare. However, the self-selection doesn’t generate bias because the intrinsic characteristics that motivate physicians to cooperate are assumed constant and hence captured in the model’s fixed effects and the variable indicating participation in GK.

We identified several mechanisms through which integrated systems create more cooperative networks. First, to meet health needs in a holistic approach, integrated healthcare pushes patients to seek care from several providers with different specialities [6,47]. This mechanism generates cooperation relationships due to a greater number of shared patients, which creates further positive externalities [6]. While this mechanism might seem counterproductive in achieving cost savings, integrated systems have shown that the synergies generated when attending to population health needs as a network more than compensate the “transaction costs” of more physician visits [5]. The benefits of this trade-off are reflected by positive results in cost savings, health system efficiency and population health [34]. The second mechanism entails positioning IPs as central actors in the regional care coordination strategy. Building on the physician-patient relationship, IPs are the starting point of the patient’s pathway through integrated healthcare. Supported by the capacities of the integrated system, IPs are the primary contact point through which patients receive the benefits of the integrated approach. Consequently, participating physicians improve their centrality in the network and can use their advantageous position to propagate best practices and innovations. Finally, integrated systems improve cooperation indirectly by advocating for better professional cooperation, providing incentives to improve system performance, incentivizing data sharing for centralized data analytics, and providing social instances where professionals in the network can interact.

Enhancing and exploiting professional relationships is a critical aspect of the integrated healthcare approach [48]. Current policy guidance supporting integrated care initiatives considers better communication, cooperation, and other forms of professional relationships as being enabled by integrated care policies [49,50,51]. However, most evaluations of integrated care focus on financial, clinical, and quality-of-life outcomes [52,53] and literature exploring improvements in professional relationships or other process-level mechanisms is scarce. Valentijn et al. [54] show that integrated care projects in primary care can effectively strengthen collaborative processes between providers. Wodskou et al. [30] and Tummers et al. [55] have similar conclusions. Research by Behrendt & Ramanuj [56] suggests that integrated care initiatives can improve professional relationships but that top-down approaches can disturb the intrinsic motivation to cooperate. On the other hand, Atwal & Caldwell [57] don’t find sufficient evidence to suggest that integrated programs enhance professional relationships. Further research should continue to examine the relationship between integrated care initiatives and effective enhancement of professional relationships, specifically to understand the characteristics that influence the desired effect in this vital process. Our article contributes to the body of evidence supporting policy guidelines towards integrated care and helps in bridging the scarcity of methodological tools to measure the correct implementation of these initiatives.

## Limitations

Data limitations prevented us from creating an impact evaluation of the causal effect GK had over regional cooperation. Instead, our design only provides observational evidence by comparing the evolution of IPs versus Non-IPs. Moreover, IPs are incentivized to cooperate with any practice in the region, independent of their participation in GK. This generates a spillover effect that underestimates the impact of GK.

Further, we were limited to data from only one insurance company. While this will most likely not bias the difference between IPs and Non-IPs, there might be cooperation relationships that we are not considering. Finally, even though higher than the critical 5% error, the model for EV-centrality presented concerning Hansen-Sargan tests results (Roodman recommends values 0.1 and higher [45]), indicating a higher probability for the instruments not to be exogenous.

## Conclusion

Our performance assessment of an integrated care program shows an increasingly cooperative network over time, led by physician practices participating actively in the integrated system. Moreover, we provide a comprehensive design for systematically monitoring cooperation in a regional healthcare network using claims data. As demonstrated in this paper, healthcare authorities can use the methodology to assess the system’s performance in improving professional cooperation, a key concept for the successful implementation of IHSs.

## Data Accessibility Statement

Data is not available for public use. Analysis was conducted during 2020 in the context of a partnership between data owners and OptiMedis AG, employer of authors NL, SW and OG.

**Code availability:** Upon request.

## Additional File

The additional file for this article can be found as follows:

10.5334/ijic.6519.s1 Appendix. Appendix 1 to 4.

## Footnotes

Subtracting the sample mean from each observation so that they are mean zero.
