# Supplementary material for: Cooperation Improvement in an Integrated Healthcare Network: A Social Network Analysis
Source: Int J Integr Care. 2023 Jun 26;23(2):32. doi: 10.5334/ijic.6519 (PMC10312245; doi:10.5334/ijic.6519)
Supplement: Appendix. — Appendix 1 to 4. [file ijic-23-2-6519-s1.pdf]

Appendix.

1. Data transformation

We converted the claims database into an adjacency matrix for network analysis(37). The adjacency matrix is unipartite and represents the number of patients shared by pairs of physicians.

$$\textbf{Claims data}$$

$$1) \quad \begin{array}{cc} Patient & Doctor \\ x & A \\ x & B \\ y & A \\ y & C \end{array} \rightarrow (two - way\ table) \rightarrow \begin{array}{cccc} Patient/Doctor & A & B & C \\ x & 1 & 1 & 0 \\ y & 1 & 0 & 1 \end{array}$$

$$\textbf{Bipartite A. matrix}$$

$$2) \quad bipartite^T * bipartite = \begin{array}{cccc} Doctor & A & B & C \\ A & 2 & 1 & 1 \\ B & 1 & 1 & 0 \\ C & 1 & 0 & 1 \end{array}$$

$$\textbf{Unipartite A. matrix}$$

2. Estimator at node level.

Generalized Methods of Moments estimation.

The standard GMM estimator (1) uses first difference of the regression equation to eliminate the individual effects. Next, lags of the dependent variable extended further back to the lag predicting the dynamic nature of the model are used as instruments for differenced lags of the dependent variable.

In practice, the standard GMM estimator consists in solving the unknown parameter vector (θ) by equating the theoretical moments with their empirical estimates. Where N is the size of a random sample, θ is the unknown parameter vector and θ_0 is the true value of θ, the model is defined when we consider the moment conditions m such that:

$$E(m(y_t;\theta_0)) = 0$$

Understanding $\widehat{m}(y_t,\theta)$ as the sample (empirical) estimate of $E(m(y_t;\theta))$ based on N independent samples of y_t:

$$\widehat{m}(y_t,\theta) = \left(\frac{1}{N}\sum_{t=1}^{N} m(y_t;\theta)\right)$$

So, the GMM consists in finding $\hat{\theta}$ such that:

$$\widehat{m}(y_t,\hat{\theta}) = 0$$

If the system is over-identified, meaning the length of the vector of moments conditions is larger than the length of the parameter vector, then the GMM estimator is defined by the minimization of the distance measure of $\widehat{m}\ (y_t,\theta)$.

$$\hat{\theta} = arg\ min_b\ \hat{m}(y_t, \theta)'\ W\hat{m}\ (y_t, \theta)$$

Where W a positive definite weighting matrix. The two steps approach is used in the presence of heteroscedasticity and correlation to secure an optimal weighting matrix $W(\hat{\theta})$; i.e., a consistent estimate of the inverse of the asymptotic covariance matrix of $m(\hat{\theta})$, by exploiting the residuals of the first step, that utilizes a suboptimal W.

$$W(\hat{\theta}) = \left(\frac{1}{N}\sum_{i=1}^{N} m_i(\hat{\theta})\, m_i(\hat{\theta})'\right)^{-1}$$

$$\hat{\hat{\theta}} = arg\ min_b\ \hat{m}(y_t, \theta)'\ W(\hat{\theta})\ \hat{m}(y_t, \theta)$$

The GMM system estimator is an alternative that corrects the bias generated by standard GMM in short, unbalanced panels with large number of units where the lagged variables are not good instruments of the variables in first differences. The method combines the standard set of transformed equations in first differences with an additional set of equations in levels. The first set of transformed equations uses the lag levels as instruments and the level equation uses the lagged first differences as instruments. Their validity is based on the following moment condition:

$$E\left[(FE_i + \varepsilon_{it})\Delta y_{i,t-z}\right] = 0\ for\ z = \{1,2\}$$

3. Assumption testing for dynamic panel estimations

3.1 Stationarity of the node level indicators panel (Using tseries::adf.test in R):

Augmented Dickey-Fuller Test<br>Alternative hypothesis: stationary			
data	Degree	EV-Centrality ranking	BE-Centrality ranking
Dickey-Fuller	-13.084	-16.62	-17.183
Lag order	2	1	1
p-value	0.01	0.01	0.01

3.2 Multicollinearity in dynamic panel model (examined with car::vif in R).

Variance Inflation factors<br>Rule of thumb: VIF over 5 is problematic	
Charlson	1.019
Total number of patients	1.124
Year	1.009
Year*IP	1.113

3.3 Normality in error distributions (using qqnorm in R)

For degree:

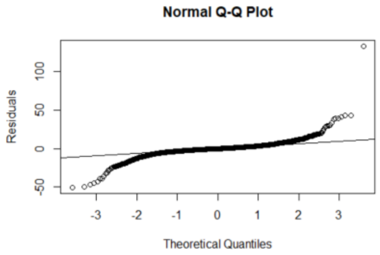

For EV-Centrality ranking:

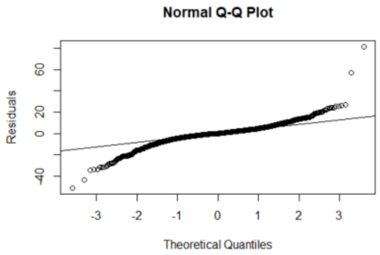

For BE-Centrality ranking:

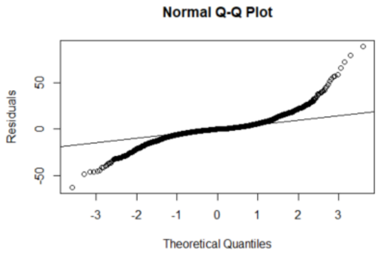

3.4 Heteroskedasticity (Using Breusch-Pagan test, lmtest::bptest in R)

Breusch-Pagan test<br>Alternative hypothesis: heteroskedasticity			
data	Degree	EV-Centrality ranking	BE-Centrality ranking
BP	13484	5279.2	3360.2
df	4	4	4
p-value	< 2.2e-16	< 2.2e-16	< 2.2e-16

We used robust standard errors(2) to deal with the presented heteroskedasticity.

4. Sensitivity analysis for different threshold of shares patients to identify a meaningful professional relationship.

Model: Degree.

Threshold	9	3	6	12	15
	Coefficients (SE)	Coefficients (SE)	Coefficients (SE)	Coefficients (SE)	Coefficients (SE)
lag(y, 1)	0.52 ***<br>(0.12)	0.78***<br>(0.09)	0.60***<br>(0.10)	0.51**<br>(0.16)	0.51*<br>(0.22)
lag(y, 2)	0.04<br>(0.14)	-0.07<br>(0.09)	0.01<br>(0.12)	-0.02<br>(0.12)	-0.23<br>(0.21)
year	1.70e-04<br>(5.39e-04)	1.25e-03<br>(1.48e-03)	8.63e-04<br>(8.80e-04)	5.26e-04<br>(6.16e-04)	3.87e-04<br>(6.68e-04)
year*IP	1.64e-03·<br>(9.08e-04)	6.86e-04<br>(2.82e-03)	2.38e-03*<br>(1.11e-03)	1.34e-03·<br>(7.44e-04)	1.17e-04<br>(1.08e-03)
Charlson Score	1.73**<br>(0.61)	3.26**<br>(1.18)	2.42**<br>(0.75)	1.14*<br>(0.46)	1.18*<br>(0.50)
Total N° patients	1.93e-02 *<br>(8.83e-03)	0.02<br>(0.01)	0.02*<br>(8.80e-04)	0.02*<br>(8.28e-03)	0.03*<br>(0.01)
#Obs	3067	3067	3067	2811	2676
nodes	602	602	602	559	537
Length of res. vector	4493	3675	4493	4181	3346
Hansen-Sargan test:	chisq(68) 58.95 (p-value: 0.78)	chisq(54) 69.06 (p-value: 0.08)	chisq(65) 74.00 (p-value: 0.21)	chisq(50) 42.35 (p-value: 0.77)	chisq(26) 22.84 (p-value: 0.64)
Autocorrelation test (1)	normal -1.98 (p-value: 0.04)	normal -4.33 (p-value< 0.05)	normal -2.38 (p-value< 0.05)	normal -1.99 (p-value: 0.05)	normal -2.25 (p-value: 0.03)
Autocorrelation test (2)	normal 1.04 (p-value: 0.30)	normal 1.46 (p-value: 0.15)	normal 1.33 (p-value: 0.18)	normal 1.57 (p-value: 0.12)	normal 1.91 (p-value: 0.06)
Wald test for coefficients	chisq(6) 7946.341 (p-value: < 2.22e-16)	chisq(6) 12609.57 (p-value: < 2.22e-16)	chisq(6) 8532.27 (p-value: < 2.22e-16)	chisq(6) 6525.30 (p-value: < 2.22e-16)	chisq(6) 3210.03 (p-value: < 2.22e-16)
R2	0.89	0.91	0.89	0.89	0.88
Instruments	Lags 4:11	Lags 6:14	Lags 4:10	Lags 4:7	6th lag

Significance: *** = p < 0.001; ** = p < 0.01; * = p < 0.05; · = p < 0.1; SE: Standard Errors

Model: EV-centrality ranking

Threshold	9	3	6	12	15
	Coefficients (SE)	Coefficients (SE)	Coefficients (SE)	Coefficients (SE)	Coefficients (SE)
lag(y, 1)	0.59***<br>(-0.12)	0.69***<br>-0.11	0.70***<br>(0.11)	0.70***<br>(0.13)	0.80***<br>(0.13)
lag(y, 2)				0.16<br>(0.22)	0.18<br>(0.24)
lag(y, 3)				-0.24<br>(0.28)	3.58e-03<br>(0.26)
year	3.96e-03*<br>(1.65e-03)	2.44e-03·<br>(1.31e-03)	2.39e-03<br>(1.7e-03)	3.58e-03<br>(2.07e-03)	3.57e-03·<br>(1.85e-03)
year*IP	3.27e-03·<br>1.82e-03	2.02e-03<br>(1.72e-03)	1.91e-03<br>(1.73e-03)	2.59e-03*(p value 0.10)*<br>(1.66e-03)	2.61e-03·<br>(1.52e-03)
Charlson Score	2.89***<br>(0.78)	2.37**<br>(0.77)	2.39**<br>(0.77)	2.41*<br>(1.03)	1.92*<br>(0.87)
Total N° patients	0.01<br>(5.38e-03)	9.32e-03<br>(6.60e-03)	0.01<br>6.76e-03	0.01<br>(7.90e-03)	8.51e-03<br>(6.23e-03)
#Obs	3067	3067	3067	2811	2676
nodes	602	602	602	559	537
Length of res. vector	3675	2406	2406	2285	2200
Hansen-Sargan test:	chisq(52) 67.45 (p-value: 0.08)	chisq(39) 34.37 (p-value: 0.68)	chisq(42) 34.10 (p-value: 0.80)	chisq(34) 32.31 (p-value: 0.55)	chisq(34) 28.02 (p-value: 0.76)
Autocorrelation test (1)	normal -5.28 (p-value: 1.32e-07)	normal -4.58 (p-value: 4.61e-06)	normal -4.61 (p-value: 4.11e-06)	normal -2.45 (p-value: 0.01)	normal -2.17 (p-value: 0.03)
Autocorrelation test (2)	normal 1.89 (p-value: 0.06)	normal 0.84 (p-value: 0.4)	normal 0.91 (p-value: 0.37)	normal -0.37 (p-value: 0.71)	normal -0.77 (p-value: 0.43)
Wald test for coefficients	chisq(5) 16151.79 (p-value: < 2.22e-16)	chisq(5) 16093.52 (p-value: < 2.22e-16)	chisq(5) 15758.24 (p-value: < 2.22e-16)	chisq(7) 14187.57 (p-value: < 2.22e-16)	chisq(7) 14031.5 (p-value: < 2.22e-16)
R2	0.92		0.93	0.91	0.91
Instruments	Lags 6:11	Lags 9,11,12,13	Lags 9,11,12,13	Lags 9:10	Lags 9:10

Significance: *** = p < 0.001; ** = p < 0.01; * = p < 0.05; · = p < 0.1; SE: Standard Errors

Model: BE-centrality ranking

Threshold	9	3	6	6	12	15
	Coefficients (SE)	Coefficients (SE)	Coefficients (SE)	Coefficients (SE)	Coefficients (SE)	Coefficients (SE)
lag(y, 1)	0.32 *** (0.05)	0.57*** (0.09)	0.37** (0.06)	0.37* (0.17)	0.41*** (0.08)	0.46*** (0.07)
lag(y, 2)		0.20** (0.07)			0.14* (0.06)	
year	1.05e-02*** (1.21e-03)	2.38e-03 (1.54e-03)	9.08e-03*** (1.41e-03)	8.86e-03** (2.76e-03)	6.09e-03** (1.91e-03)	7.22e-03*** (1.31e-03)
year*IP	4.56e-03*** (7.94e-04)	1.91e-03* (9.10e-04)	5.17e-03*** (1.22e-03)	4.34e-03*(p value 0.12)* (2.81e-01)	2.21e-03** (8.51e-04)	2.69e-03*** (8.13e-04)
Charlson Score	2.31*** (0.61)	1.32* (0.54)	2.36*** (0.71)	2.71* (1.11)	1.80** (0.56)	1.77** (0.64)
Total N° patients	1.48e-02*** (2.01e-03)	6.02e-03 (2.48e-03)	0.01*** (2.57e-03)	0.01· (7.71e-03)	0.01** (3.78e-03)	0.01*** (1.798e-03)
#Obs	3067	3067	3067	3067	2811	2676
nodes	602	602	602	602	559	537
Length of res. vector	4894	4100	4894	1525	4499	4479
Hansen-Sargan test:	chisq(81) 74.91 (p-value: 0.67)	chisq(56) 58.83 (p-value: 0.37)	chisq(81) 102.25 (p-value: 0.06)	chisq(45) 35.50 (p-value: 0.84)	chisq(44) 53.78 (p-value: 0.15)	chisq(87) 101.99 (p-value: 0.13)
Autocorrelation test (1)	normal -6.15 (p-value: 7.89e-10)	normal -3.93 (p-value: 8.39e-05)	normal -4.74 (p-value: 2.11e-06)	normal -2.84 (p-value: 4.50e-03)	normal -3.69 (p-value: 2.28e-04)	normal -4.57 (p-value: 4.89e-06)
Autocorrelation test (2)	normal 0.91 (p-value: 0.37)	normal -0.61 (p-value: 0.54)	normal 0.78 (p-value: 0.43)	normal 1.23 (p-value: 0.22)	normal 0.47 (p-value: 0.64)	normal 0.54 (p-value: 0.59)
Wald test for coefficients	chisq(5) 10526.45 (p-value: < 2.22e-16)	chisq(6) 28368.16 (p-value: < 2.22e-16)	chisq(5) 9814.532 (p-value: < 2.22e-16)	chisq(5) 4208.49 (p-value: < 2.22e-16)	chisq(6) 18987.58 (p-value: < 2.22e-16)	chisq(5) 10654.09 (p-value: < 2.22e-16)
R2	0.83	0.89	0.84	0.87	0.84	0.84
Instruments	Lags 3:14	Lags 5:10	Lags 3, 11:14	Lags 11:14	Lags 3, 5	Lags 2, 4, 10

Significance: *** = p < 0.001; ** = p < 0.01; * = p < 0.05; · = p < 0.1; SE: Standard Errors

References

1. **Arellano M, Bond S.** Some Tests of Specification for Panel Data: Monte Carlo Evidence and an Application to Employment Equations. *Rev Econ Stud*. 1991 Apr; 58(2): 277. DOI: https://doi.org/10.2307/2297968
2. **Freedman DA.** On The So-Called "Huber Sandwich Estimator" and "Robust Standard Errors." *Am Stat*. 2006 Nov; 60(4): 299–302. DOI: https://doi.org/10.1198/000313006X152207
